# Safety of Transvaginal Specimen Retrieval in Total Laparoscopic Hysterectomy for Nulliparous Women: A Retrospective Study

**DOI:** 10.7759/cureus.99020

**Published:** 2025-12-12

**Authors:** Noritoshi Aimoto, Takashi Matsumoto, Yonosuke Tsuda, Hosokawa Yumi

**Affiliations:** 1 Gynecology, Osaka Central Hospital, Osaka, JPN

**Keywords:** in-bag morcellation, nulliparous women, surgical safety, total laparoscopic hysterectomy, transvaginal specimen retrieval

## Abstract

Total laparoscopic hysterectomy (TLH) is a widely accepted minimally invasive procedure for benign gynecologic diseases. Transvaginal retrieval avoids additional abdominal incisions but may be technically challenging in nulliparous women because of narrower vaginal dimensions and less distensible tissues, particularly in cases with a large uterus. We retrospectively analyzed 368 nulliparous patients who underwent TLH at our institution between February 2021 and August 2025. Among them, 267 underwent transvaginal retrieval and 101 underwent transabdominal retrieval, with all cases performed using contained in-bag morcellation. In the high-difficulty subgroup defined as uterine weight ≥500 g (n = 147), 62 underwent transvaginal and 85 underwent transabdominal retrieval. No conversion from transvaginal to transabdominal extraction was required. Compared with the abdominal route, transvaginal retrieval was associated with significantly shorter operative time (median 203.5 vs. 207.0 minutes, p = 0.016) and retrieval time (28.5 vs. 34.0 minutes, p = 0.024), while blood loss did not differ significantly (25 vs. 25 ml, p = 0.142). Retrieval efficiency (uterine weight ÷ retrieval time) tended to be higher in the transabdominal group (median 25.69 vs. 24.14 g/min, p = 0.083), but the difference was not statistically significant. No major complications occurred, and only minor events such as vaginal wall lacerations and vaginal cuff infections were observed (vaginal wall laceration: 1/62 [1.6%] vs. 2/85 [2.4%]; vaginal cuff infection: 1/62 [1.6%] vs. 1/85 [1.2%] in the transvaginal and transabdominal groups, respectively); all were managed conservatively or with simple intraoperative repair. These findings suggest that even in nulliparous women with large uteri, transvaginal retrieval during TLH using contained in-bag morcellation can be considered a safe and effective first-line option for specimen removal when intraoperative conditions permit, potentially providing superior cosmetic results and reducing surgical morbidity without increasing perioperative risks. Prospective, multicenter studies are warranted to validate these results.

## Introduction

Total laparoscopic hysterectomy (TLH) has become a widely accepted minimally invasive procedure for benign gynecologic diseases, offering advantages including reduced blood loss, shorter hospitalization, and faster postoperative recovery compared with traditional open surgery [1,2]. A key component of the procedure is the extraction of the uterine specimen. Transvaginal specimen retrieval avoids additional abdominal incisions, such as a minilaparotomy, thereby further enhancing the cosmetic and minimally invasive advantages of the surgery. At our institution, transvaginal retrieval is preferred when intraoperative assessment indicates that it can be performed safely.

However, in nulliparous women, narrower vaginal dimensions and less distensible tissues often make extraction technically challenging, particularly in cases with a large uterus, raising concerns regarding prolonged operative duration and potential iatrogenic vaginal injury [3-5]. The method of specimen removal has also been a subject of debate. In addition to these anatomical limitations, evolving safety concerns regarding tissue extraction methods, particularly morcellation, have influenced surgical practice. In 2014, the U.S. Food and Drug Administration (FDA) issued a safety communication advising against the use of laparoscopic power morcellators for hysterectomy or myomectomy due to the risk of disseminating occult malignant tissue [6]. This prompted the development and widespread adoption of contained in-bag morcellation techniques, which aim to balance minimal invasiveness with oncologic safety [7,8].

Most previous studies examining specimen retrieval routes have included multiparous women or mixed cohorts, and dedicated data focusing exclusively on nulliparous women are limited. Given these considerations, we conducted a retrospective study limited to nulliparous women who underwent TLH to evaluate patient characteristics and surgical outcomes, aiming to clarify the safety and feasibility of transvaginal retrieval using contained in-bag morcellation, especially in high-difficulty cases.

## Materials and methods

Study design and patient population

This retrospective cohort study was conducted at our hospital. We analyzed the medical records of 688 consecutive patients who underwent TLH for benign gynecologic diseases between February 2021 and August 2025. Inclusion criteria were (1) nulliparous status, (2) undergoing TLH for a benign indication (e.g., myoma, adenomyosis, endometriosis), and (3) complete medical records available. Exclusion criteria were (1) suspected or confirmed gynecologic malignancy, (2) concurrent major surgical procedures, and (3) non-nulliparous patients. All TLH cases during the study period were enrolled consecutively, and all nulliparous patients who met the inclusion criteria (n = 368) were included in the final analysis without additional selection.

This study was approved by the Institutional Review Board of our hospital, and the requirement for informed consent was waived because of the retrospective design and use of anonymized patient data. The study was conducted in accordance with the principles of the Declaration of Helsinki and relevant national guidelines for research involving human subjects.

Surgical procedure and specimen retrieval

All TLH procedures were performed under general anesthesia using a standardized four-port laparoscopic approach. The operating surgeon decided the specimen retrieval route intraoperatively based on a comprehensive assessment of vaginal distensibility, uterine size, consistency, and mobility. When transvaginal retrieval was judged to be unsafe or inappropriate, the abdominal route was chosen. Contained in-bag morcellation was used in all cases to minimize the risk of tissue dissemination.

**Figure 1:**
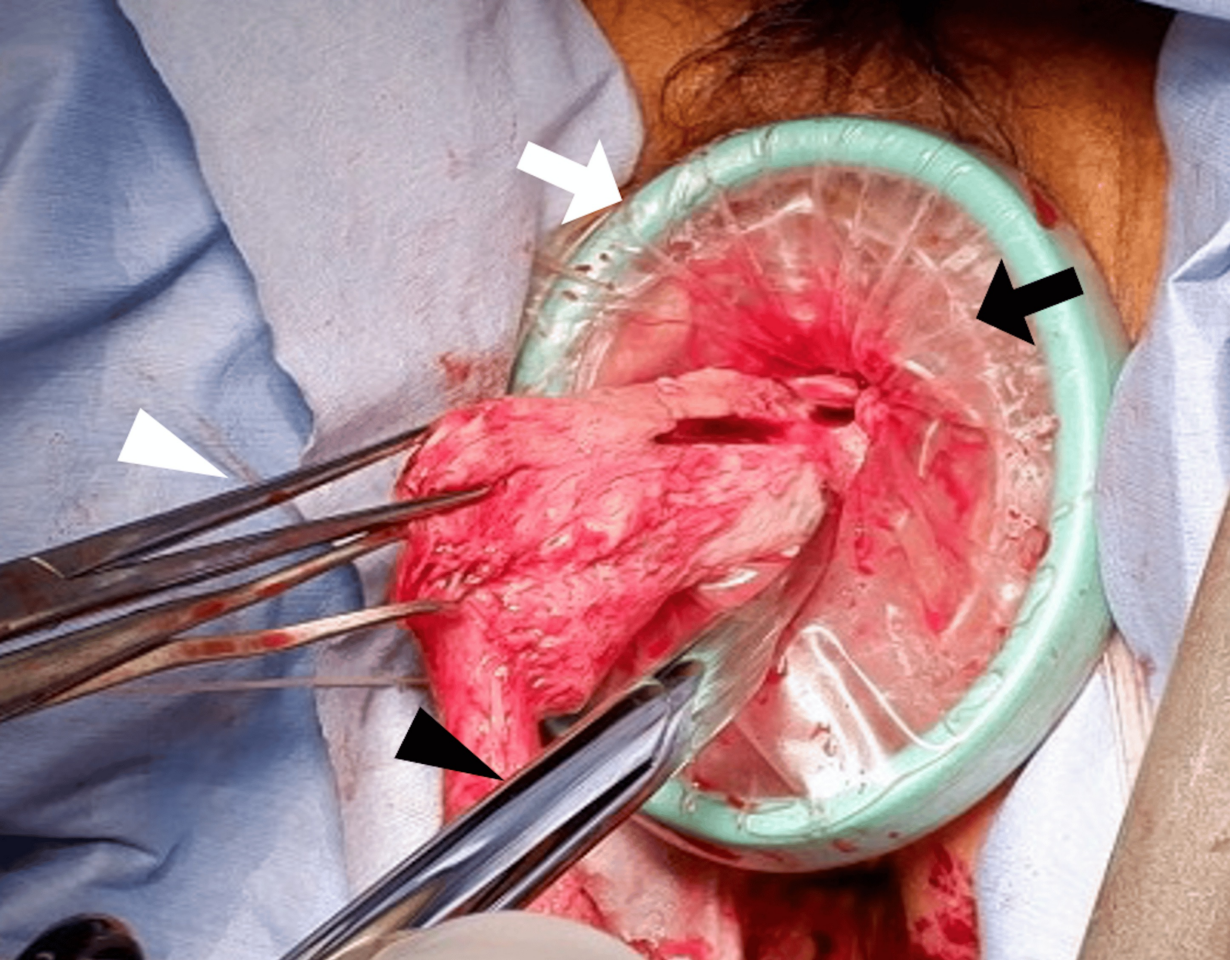
Intraoperative view of transvaginal specimen retrieval. White triangle: Martin forceps; black triangle: Supercut scissors; white arrow: Smart Retractor Free Access; black arrow: Extraction Bag.

For transvaginal retrieval, the uterus was inserted into a retrieval bag (EZ Purse; Hakko Co., Ltd., Tokyo, Japan; or Extraction Bag; Karl Storz SE & Co. KG, Tuttlingen, Germany), the Smart Retractor Free Access (Top Corporation, Tokyo, Japan) was attached to the vaginal stump to obtain visualization and protect the vaginal wall, and morcellation was performed using Martin forceps, a scalpel, and Supercut scissors (Figure 1). A single senior surgeon performed all transvaginal retrievals to ensure consistency of technique.

For transabdominal retrieval, the uterus was placed in a retrieval bag (MorSafe; Adachi Co., Ltd., Tokyo, Japan; or Isolation Bag; Karl Storz SE & Co. KG, Tuttlingen, Germany) and morcellated with a Rotocut G1 (Karl Storz SE & Co. KG, Tuttlingen, Germany). Transabdominal retrievals were performed by board-certified gynecologic surgeons or fellows under direct supervision, all of whom followed the same institutional protocol for contained in-bag morcellation.

Data collection and definitions

We collected the following data from electronic medical records and surgical video recordings: patient demographics (age, body mass index [BMI]), surgical indication, uterine weight (measured postoperatively), retrieval route, total operative time (from skin incision to skin closure), amount of blood loss, and perioperative complications.

Transvaginal retrieval time was defined as the interval from insertion of the retrieval bag through the umbilical port until removal through the vagina, and transabdominal retrieval time was defined as the interval from insertion of the bag through the left lower abdominal port until removal through the abdominal wall. Time spent on unrelated hemostasis or suturing was excluded. Retrieval efficiency was calculated as uterine weight (g) divided by retrieval time (min). A high-difficulty subgroup was defined as patients with a uterine weight of ≥500 g. Perioperative complications were graded according to the Clavien-Dindo classification [9], with major complications defined as grade III or higher.

To ensure data accuracy, information extracted from electronic medical records was cross-checked against operative reports and surgical video recordings by two investigators, and discrepancies were resolved by consensus.

Statistical analysis

All statistical analyses were performed using EZR (Easy R, version 1.62; Jichi Medical University Saitama Medical Center, Saitama, Japan), a graphical user interface for R (R Foundation for Statistical Computing, Vienna, Austria) [10]. Continuous variables were assessed for normality and are presented as median and interquartile range (IQR). Comparisons between the transvaginal and transabdominal retrieval groups were performed using the Mann-Whitney U test for continuous variables. Categorical variables, such as complication rates, were compared using the chi-square test or Fisher’s exact test, depending on expected frequencies. Statistical significance was defined as a p-value of <0.05. Cases with missing data on key variables were excluded from the corresponding analyses; no imputation was performed.

Use of tools and scales

All proprietary surgical instruments and devices mentioned (e.g., EZ Purse, Smart Retractor Free Access, Rotocut G1) are commercially available and were used in accordance with the manufacturers' instructions. The Clavien-Dindo classification [9], a standard tool for grading surgical complications, is freely available for clinical and research use and was applied as originally described. The Pelvic Organ Prolapse Quantification (POP-Q) system [11], mentioned in the Discussion, is also an established, freely available clinical standard.

## Results

Among 368 nulliparous women, 267 underwent vaginal retrieval and 101 underwent transabdominal retrieval. We defined the high-difficulty group as nulliparous patients with uterine weight ≥500 g, which included 147 cases (62 vaginal, 85 abdominal). No patient required conversion from transvaginal to transabdominal retrieval.

Patient characteristics showed no significant differences in age or BMI between the groups. The median uterine weight was 700 g (IQR 578-887) in the transvaginal group and 875 g (IQR 729-1045) in the transabdominal group, which was significantly higher in the latter (p < 0.001; Table 1).

**Table 1:** Patient characteristics (high-difficulty subgroup, uterine weight ≥500 g). Values are presented as median (interquartile range). Statistical analysis performed using Mann–Whitney U test.

Variable	Transvaginal group, n=62 Median (IQR)	Transabdominal group, n=85 Median (IQR)	U-value	p-value
Age (years)	47.0 (44.2–49.0)	47.0 (45.0–50.0)	2845.0	0.806
BMI (kg/m²)	22.2 (20.4–24.7)	23.1 (21.0–26.3)	2425.5	0.105
Uterine weight (g)	700 (578–887)	875 (729–1045)	1765.5	<0.001

Operative outcomes demonstrated that operative time was significantly shorter in the transvaginal group (203.5 min, IQR 173.2-214.8) compared with the transabdominal group (207.0 min, IQR 190.0-255.0; p = 0.016). Retrieval time was also shorter in the transvaginal group (28.5 min, IQR 24.0-37.5) compared with the transabdominal group (34.0 min, IQR 28.0-43.0; p = 0.024). Estimated blood loss did not differ significantly between the groups (25 ml, IQR 15-30 vs. 25 ml, IQR 15-35; p = 0.142). Retrieval efficiency tended to be higher in the transabdominal group (25.69 g/min, IQR 22.11-30.71) than in the transvaginal group (24.14 g/min, IQR 20.72-27.94), but the difference was not significant (p = 0.083; Table 2).

**Table 2:** Surgical outcomes (high-difficulty subgroup, uterine weight ≥500 g). Retrieval time = time required solely for uterine extraction after insertion of the retrieval bag. Retrieval efficiency = uterine weight divided by retrieval time. Values are presented as median (interquartile range). Statistical analysis was performed using the Mann–Whitney U test.

Variable	Transvaginal group, n=62 Median (IQR)	Transabdominal group, n=85 Median (IQR)	U-value	p-value
Operative time (min)	203.5 (173.2–214.8)	207.0 (190.0–255.0)	2185.0	0.016
Retrieval time (min)	28.5 (24.0–37.5)	34.0 (28.0–43.0)	2542.0	0.024
Blood loss (ml)	25 (15–30)	25 (15–35)	2572.0	0.142
Retrieval efficiency (g/min)	24.14 (20.72–27.94)	25.69 (22.11–30.71)	2225.0	0.083

No major complications (Clavien-Dindo grade ≥III) occurred. Minor complications included one vaginal wall laceration in the transvaginal group and two in the transabdominal group, as well as one vaginal cuff infection in each group. These corresponded to vaginal wall lacerations in 1/62 (1.6%) vs. 2/85 (2.4%) and vaginal cuff infections in 1/62 (1.6%) vs. 1/85 (1.2%) in the transvaginal and transabdominal groups, respectively. All minor complications were managed successfully with intraoperative repair or conservative treatment.

## Discussion

This study demonstrated that transvaginal retrieval with contained in-bag morcellation during TLH can be safely performed even under challenging conditions, namely in nulliparous women with large uteri (≥500 g). In the high-difficulty subgroup, vaginal retrieval resulted in significantly shorter operative and retrieval times compared with the abdominal route, without an increase in blood loss or major perioperative complications.

Because surgeons determined the retrieval route intraoperatively, selection bias may exist: cases with lighter uterine weight, mild adhesions, and greater uterine mobility were more likely to undergo vaginal retrieval. Indeed, uterine weight was significantly higher in the abdominal group. Although all TLH cases during the study period were enrolled consecutively and all eligible nulliparous women were included in the analysis, this surgeon-driven route selection remains an inherent limitation of our retrospective design.

We defined a large uterus as ≥500 g, consistent with previous reports, and prior studies have demonstrated that uterine weights above this threshold are associated with an increased risk of intraoperative complications [12,13]. Nevertheless, retrieval difficulty depends not only on weight but also on uterine morphology and consistency. We did not formally subclassify patients according to uterine shape (e.g., transversely enlarged uterus or single dominant leiomyoma); such cases were assigned to each group based on intraoperative assessment and the chosen retrieval route. Incorporating uterine morphological classifications and assessments of tissue firmness may allow a more accurate evaluation of difficulty. For example, classifications based on uterine enlargement patterns (e.g., upward vs. lateral enlargement) have been proposed as useful predictors of TLH operative time [14,15]. Future studies that integrate weight, morphology, and consistency into a comprehensive difficulty assessment may refine patient selection for each retrieval route.

Vaginal dimensions and tissue distensibility also strongly influence procedural feasibility, but no standardized objective method exists to measure these factors other than the POP-Q system used for pelvic organ prolapse [11]. Future research may introduce objective assessments, such as MRI- or ultrasound-based measurements of vaginal diameter, or the use of mechanical dilators, to establish more precise selection criteria and thresholds that indicate when transvaginal retrieval is likely to be safe and efficient.

To ensure safe vaginal retrieval, several technical considerations are important. Applying the Smart Retractor Free Access to the vaginal stump provides uniform exposure and protects the vaginal wall, thereby reducing the risk of laceration. During morcellation, rotating the uterus within the bag and initiating the incision from the cervix can improve efficiency, while placing gauze beneath the uterus can provide stability. In our series, no case required conversion to abdominal retrieval, suggesting that standardization of techniques and accumulation of surgical experience may increase the upper limit of extractable uterine weight via the vaginal route.

This study has several limitations. First, it was a retrospective, single-center study, which may limit the generalizability of our findings. Second, the choice of retrieval route was left to the intraoperative judgment of the surgeon, introducing potential selection bias. Third, all transvaginal retrievals were performed by a single experienced surgeon, whereas multiple surgeons with varying levels of experience performed the transabdominal retrievals, raising the possibility of performance bias despite standardized institutional protocols. Fourth, we did not evaluate long-term outcomes such as vaginal healing, pelvic floor function, or sexual function, which may be relevant to route selection. Finally, we did not systematically classify uterine morphology or quantify vaginal distensibility, both of which likely influence procedural difficulty.

Nonetheless, our study has important strengths. It focuses on a relatively large cohort of nulliparous women, a group for whom evidence remains limited. All procedures were performed using a standardized laparoscopic approach with contained in-bag morcellation, and we collected detailed operative data, including retrieval time and retrieval efficiency, allowing a nuanced comparison between routes. These features support the internal validity of our findings and provide a useful foundation for future prospective work.

## Conclusions

We demonstrated that surgeons can safely perform transvaginal retrieval with contained in-bag morcellation during TLH, even in nulliparous women with large uteri (≥500 g). In our cohort, this approach was associated with shorter operative and retrieval times compared with the abdominal route, without an increase in blood loss or major complications. Our findings suggest that transvaginal retrieval with contained in-bag morcellation can be considered a safe and effective first-line option for specimen removal in nulliparous women when intraoperative conditions permit.

Standardizing surgical techniques, using vaginal wall protection devices, and developing objective preoperative indicators, such as uterine weight, morphology, consistency, and vaginal distensibility, may help establish a practical algorithm for optimal route selection. Further prospective, multicenter studies are warranted to validate these results and to clarify the long-term functional outcomes of each retrieval route.

## References

[1] Nieboer TE, Johnson N, Lethaby A (2009). Surgical approach to hysterectomy for benign gynaecological disease. Cochrane Database Syst Rev.

[2] Johnson N, Barlow D, Lethaby A, Tavender E, Curr L, Garry R (2005). Methods of hysterectomy: systematic review and meta-analysis of randomised controlled trials. BMJ.

[3] Agostini A, Bretelle F, Cravello L, Maisonneuve AS, Roger V, Blanc B (2003). Vaginal hysterectomy in nulliparous women without prolapse: a prospective comparative study. BJOG.

[4] Chikazawa K, Imai K, Ko H, Ichi N, Misawa M, Kuwata T (2022). Risk factors associated with perineal and vaginal lacerations and vaginal removal in total laparoscopic hysterectomy. Gynecol Minim Invasive Ther.

[5] Sirota I, Tomita SA, Dabney L, Weinberg A, Chuang L (2019). Overcoming barriers to vaginal hysterectomy: an analysis of perioperative outcomes. J Turk Ger Gynecol Assoc.

[6] (2020). U.S. Food and Drug Administration. UPDATE: Perform Only Contained Morcellation When Laparoscopic Power Morcellation Is Appropriate: FDA Safety Communication. https://www.fda.gov/medical-devices/safety-communications/update-perform-only-contained-morcellation-when-laparoscopic-power-morcellation-appropriate-fda.

[7] Taylan E, Sahin C, Zeybek B, Akdemir A (2017). Contained morcellation: review of current methods and future directions. Front Surg.

[8] Steller C, Cholkeri-Singh A, Sasaki K, Miller CE (2017). Power morcellation using a contained bag system. JSLS.

[9] Dindo D, Demartines N, Clavien PA (2004). Classification of surgical complications: a new proposal with evaluation in a cohort of 6336 patients and results of a survey. Ann Surg.

[10] Kanda Y (2013). Investigation of the freely available easy-to-use software 'EZR' for medical statistics. Bone Marrow Transplant.

[11] Bump RC, Mattiasson A, Bø K (1996). The standardization of terminology of female pelvic organ prolapse and pelvic floor dysfunction. Am J Obstet Gynecol.

[12] Louie M, Strassle PD, Moulder JK, Dizon AM, Schiff LD, Carey ET (2018). Uterine weight and complications after abdominal, laparoscopic, and vaginal hysterectomy. Am J Obstet Gynecol.

[13] Hillis SD, Marchbanks PA, Peterson HB (1996). Uterine size and risk of complications among women undergoing abdominal hysterectomy for leiomyomas. Obstet Gynecol.

[14] Uccella S, Kho RM, Garzon S, Casarin J, Zorzato PC, Ghezzi F (2021). The Large Uterus Classification System: a prospective observational study. BJOG.

[15] Hurni Y, Fung H, Simonson C (2024). Impact of uterine weight and shape on vNOTES hysterectomy: analysis of 238 consecutive cases. J Minim Invasive Gynecol.

